# Supplementary material for: Patterns of Intron Gain and Loss in Fungi
Source: PLoS Biol. 2004 Nov 30;2(12):e422. doi: 10.1371/journal.pbio.0020422 (PMC532390; doi:10.1371/journal.pbio.0020422)
Supplement: Table S1 — Also available at http://genes.mit.edu/NielsenEtAl/. (4.3 MB ZIP). [file pbio.0020422.st001.zip › NielsenEtAl/html/138.html]

AN8701.1.NCU02112.1.MG02569.1.FG08557.1


```
 CLUSTAL W (1.82) Multiple Sequence Alignments - Introns Inserted


Sequence 1: NCU02112.1	1808 aa
Sequence 2: FG08557.1	1457 aa
Sequence 3: MG02569.1	1671 aa
Sequence 4: AN8701.1	1462 aa
Alignment Length: 1946 aa
Number Identitical Residues: 348 aa
Alignment Score (without introns) 22483


MG02569.1 	MPSAYDQKKSDSAKKGNTVKTAFDSESFDADWTIHVPGLVGSATISPSGRDVALAS~PEG
NCU02112.1	MP----SVFDSPPKKAKTIKSAFDSESFDADLTIHVDGLVGSATISPSGRDVALAS~PEG
FG08557.1 	MPSF--DQVTDAPKKGKVIKSAYDSESFESDGTIHVDGLVGSATISPAGRDVALAS2PDG
AN8701.1  	---------MSDSNEPKPLASAFDSPTFGEDSSFHVDQPVGSMSISPCGRDVVLAS~KEG
          	          . .:: : : :*:** :*  * ::**   *** :***.****.***  :*

MG02569.1 	LAIIDLDSPYSPPRRLSSRGLPWLVVDVQWSPFAARDYWVASTANHRCLVWNLNFRDDSA
NCU02112.1	LAIIDLDSPWNPPRRLSSHGLPWLVVDVQWSPFAARDYWVASTANHRCLVWNLNMRDDSP
FG08557.1 	LAIIDLDSPYSPPRRLRTHGLPWLVVDVQWSPFAARDYWIVSAANHRALVWNLNRREDSS
AN8701.1  	LHIIDLDSPYSPPRYLP-HHTPWEVADVQWSPFAARDYWVVSTSNQKALVWNLAMR--SY
          	* *******:.*** *  :  ** *.*************:.*::*::.*****  *  * 

MG02569.1 	SGPIEHSLQGHSRAITDINFSAHHPDLLVTCSVDGYVHNWDLRRPQQPAESFCDWTAGAT
NCU02112.1	SGAIEHSLQAHSRAITDINFSAHHPDLLASCSVDGYVHHWDLRRPRQPALSFCDWFAGAT
FG08557.1 	SGAIEHSLQGHSRAITDVNWSAHHPDLLSTCSVDGYIHAWDLRRPRQPVLTFCDWTAGAS
AN8701.1  	QNSIEHVLHAHTRAITDINFSAHHPDILATCAVDSFVHCWDLRATSRPAISFSDWFAGAT
          	...*** *:.*:*****:*:******:* :*:**.::* **** . :*. :*.** ***:

MG02569.1 	QVKFSRQDPHMLASSHDRLLHIWDERRPVEPVKTIMAHSSKIYGIDWNRTRASALVTCSL
NCU02112.1	QVKYSRQDPHVIASAHDRLLHIWDDRKTVEPLKTICAHTSKIYGIDWNRTDAPCLVTCSL
FG08557.1 	QVKYNRQASHILASAHDRWLHIWDERRSSEPLKSIKAHKSKIYGLDWNRSDPKSIVSCSI
AN8701.1  	QVKWNRQNPNVIASSHDRYLRIWDKRMGAYPIRSIVAHDTKIYGVDWNRVRPGALATCSL
          	***:.** .:::**:*** *:***.*    *:::* ** :****:****  . .:.:**:

MG02569.1 	DKSIKFWDYKNEGDAPERVIRTDFPVWRARHTPFGWGVLAMPQNEPGNLHLFDGRSREGD
NCU02112.1	DKTIKFWNYEKT-DKPERVIRTDFPVWRARHTPFGNGLLAMPQNEPGNLYLYDRQTAPEA
FG08557.1 	DKRINFWDYTAE-EPLQHTIETGFPVWRARHTPFGHGLLAIPQNEPGDLYLYDTRFNLES
AN8701.1  	DKTIKFWDYTVEDDVPEKVIKTPFPVWRARNTPFGWGVLAMPQRGNSDLHLYSRRAVEGA
          	** *:**:*   .:  ::.*.* *******:**** *:**:**.  .:*:*:. :     

MG02569.1 	PQDSREDPVFVFKGHGDHKAKEFLWRSRGGVDDNGIDSRDFQLVSWGADKELRLERMEAD
NCU02112.1	PSAS--EPVAMFSGHGNHKVKEFLWRTRGSITSEGVDEREFQLVSWGEDNELRLQKVEPD
FG08557.1 	KLTT--QPIEVWPGHGDHKAKEFLWRCRGGITDDGKDNREFQLVSWGTDNELKLQCIDPK
AN8701.1  	DPDNHLAPVHSFPGH-KGQVKEFLWRARGDVVD-GIDHREFQLVSWGTDKELRLHRVDPD
          	   .   *:  : ** . :.****** **.: . * * *:******* *:**:*. ::..

MG02569.1 	VLEKVGYVKGQPAPKDLVLTRKGATYKTFRTVDDGVHRDRWNGTMTDPRAGHTSNPLRRN
NCU02112.1	ILESVGYVKGKTVVKNLALTRKGAAYKTFRAVDDTVHRDRRTATMSDPRPGSSG-QYRKS
FG08557.1 	TFAAIGHKRGIPADPAISLTRKGATYKTFRTVDETADRDRKSATMSDHRPGTN--YHKQS
AN8701.1  	VLQRVGYEKGKSFISNLNITRKGAVYRSFRDENVGLELEE-DGTLSPGFATPVPPSSRGA
          	 :  :*: :* .    : :*****.*::**  :   . :.  .*::   .       :  

MG02569.1 	GIATGSQN---AYHKPASAWRAPSMKAKTMG-KESNRSLAQIGWMRGVTMSKRKTQTGTS
NCU02112.1	ALSYGLNS---HLRRVGPTWRGSPMKAKVNGGKSMDKSQLQIGWMKGISMTKRKSESDGP
FG08557.1 	ALTLGMRSGTTQSHRAAVAWRGPSMKAKVPG-RALDRNQSQIGWMKGIAMTKRKASTS-V
AN8701.1  	GINTISIPYAPAWIQGGNADSRIGMKSRTGLRTDTN----PIAWMRGVKISG--------
          	.:       :.   : . :     **::.      :     *.**:*: ::         

MG02569.1 	SSNRDALSPTDQDSIFGSVYHDNEWGEPDTLQDEFVRLTSQLPNVKWNHIDMDNLIINAS
NCU02112.1	GRGS-----IKDSGLFSHGAYDDDWAEPESIQDEFIHVSNRLPNVKWENIDMDALILNAS
FG08557.1 	KRHL-----SKDSGIFGHGYPDDDWAEPDTIQEELLRISHQLPKVKWDNINMDNMTLNAS
AN8701.1  	-------------------------WDIETLGDEITHVGEKFTKVAFESVDIRRRKATIS
          	                          : ::: :*: ::  ::.:* :: :::     . *

MG02569.1 	LKGPWGVSGDPIHIKVKVDIPKKYPKSKAPRFFIDKSSFMTEETRTKIQNELSLLVAQFA
NCU02112.1	LKGPWGFNDEGIFIKVKVDIPTSYPTSKAPKFYVEKTSFMPEETHEKIERELQILANQFL
FG08557.1 	LNGPWGNDGDTIFIKVRIDIPIGYPKTKAPKFFVERSSFMPAETHKKIDREIHQLADQFL
AN8701.1  	LHGPWGADGASLFIRVDIKFPPDYPKDAVPTFSVQRTTAVSDQLASKIIAELQTISETYL
          	*:**** ..  :.*:* :.:*  **.  .* * ::::: :. :   **  *:  :   : 

MG02569.1 	QHKKNSLGPAFAYLLGEVDLAGSTSFLRDLGDMDDMDDEDEDMETTGRNAAADEESSSDS
NCU02112.1	ERKQNCLEVAFSYLLGDVDLENSTTLFKNVRDLNDE----------EIEALADESSSDEE
FG08557.1 	QRKQNCLEVAFTYLLGEVDLESSTTFFKNVRDLDD-----------ELDGMADESSS-ES
AN8701.1  	SYKRGCLEGVVRYLLGESSLEESIAWILGETAETIKSP--------TNGHDGGDESSDED
          	. *:..*  .. ****: .*  * : : .       .              ..:.**.:.

MG02569.1 	ESDIPPGGSAAMSQELPEPTTGSDAPAALSGGFRQTVIPVKRTCGARFSNDGKLACFFPT
NCU02112.1	DSDVQARGSVSMSQDLTA-STELDPTATLAPTQRPIAVPTPRKCGARFSNDGRLVCFFPS
FG08557.1 	DEDIPAGGSASMSQELPP-HNELD--TALAPPNRTIVPPPPRTCGARFSHNGRLVCFFPS
AN8701.1  	EVGLSQSQDLAMSSELIR------------PVNANVMVPRARECGAVWGNNGRLVCFFPT
          	: .:    . :**.:*                      *  * *** :.::*:*.****:

MG02569.1 	KEEKARALFLVS-TDSVRERSKGEPRFAGFGRLLQNSQSP--TGRRNPDDSASDDGSGGS
NCU02112.1	KEQQALALFSISNSDAYRDRQKGEPNFAGFGRLTQDSPPP--RRRYNDDDSGSDDMSDGS
FG08557.1 	KEEKARALFTTT-MDAYRERPRGEPFFAGFGRIAHDLSPR--QRFMAEDTSASSGDSDSS
AN8701.1  	KKDKSAYSLENLGFKEMTRLSRSDNLFERFGRFETSSPGPRITGTIASTDDGASEYSDDS
          	*::::   :   . .      :.:  *  ***:  .              ..:.  *..*

MG02569.1 	DADD-SSSSSS--SDSELASMNRISFWYRQGRSKFRKSWSANESVRSSGGGTGTGTGTQA
NCU02112.1	ED---SESSSS--SDSEPSTMHKISLWHQPGGRSLKKTWSTNDSLRSSGGGTGAGTGTGT
FG08557.1 	ASGSSSSSSSS--SDSESTSMHKISLWYHPS-RQFRKTWSEDRSIRSSGGGTGVGTGTGT
AN8701.1  	EAETSSSSGSSGILSGLQHRFPTPQTWRSVGSLGMYRPRSTDNSQRSTVGGMTSGS----
          	    :*.*.**.  ..    :   . *   .   : :. * : * **: **   *:    

MG02569.1 	GTIRGSRRTATVKAKNIVSIHDMRDLIPSKQAYAKEYAIFGDGEEVCEHNAQVAERHGDK
NCU02112.1	G---ISRRRLPARPKNIIALHDLRSLLPSQKQFAQEYAIFGDGAEVCMHNAMVAENYGYH
FG08557.1 	G---TSRRR-PGRPKNLISIHDFRALHPSKEEFAREYAIFGDGAEVCEHNAKVAEKYNAR
AN8701.1  	----------SDTPQNVITIHDLSGILPAKRELAIAYRICGEVVDVCAHNAAVAFDHGYY
          	          .  .:*::::**:  : *::.  *  * * *:  :** *** **  :.  

MG02569.1 	DLMYIWRYAGLLLRSDIPLRLADDQQFPRKNANSFLVIARDAVSRFGGTSPPHE------
NCU02112.1	DLVHIWHYAAMLLRNDIPLEIHEQQ----NRKESVLVIARDASARVRDDEYAQHFGWD--
FG08557.1 	DLVDVWRYLALLLNKGVPLEVLSHN----QRRDSILVIAHDVVSRCNANSSPHDSLWDRH
AN8701.1  	ELARIWGLVKLILQQTKSLRSPPVMN---SMSRSLRIQRKDSAIDLSFDALHPDKTS---
          	:*  :*    ::*..  .*.     .   .   *. :  :*            .      

MG02569.1 	ADMFLGKVKWGQHPLVH-DFISDVFAYFEQLADIQMLAMLSCVFGDSWAKDSLAYAESHL
NCU02112.1	-SNLSGRVKWGYHPLAQ-SLIADLFNYFEKVADIQMLAMLACIFGDSSAEDGVAYAGSHL
FG08557.1 	EGALSGRVKWGKHPLAR-DFIMDLFAYFEKIADIQMLAMLSCIFSESSAADSVAYFESHL
AN8701.1  	-QNTTNVVEWGNHPFGGNLLIPALFAHFEQVGDIQMAAMLSCVLYEPNLERSPSKGE---
          	     . *:** **:  . :*  :* :**::.**** ***:*:: :.    . :      

MG02569.1 	TQPQTPLPMKAPSFSLEYFPTNPELWHQQQLQSNWQHQHHHFRSQASSAITTPRTSQTPN
NCU02112.1	PQPETPLPMKAPSFSLEYFPTDPALWHIN------------YKSQASSAITTPRTVHTP-
FG08557.1 	PQPDTPLPMKAPAFSLDYFPTDASLWNIGS-----------GRSYTNSAITTPMTVHTP-
AN8701.1  	---LPPEQADHFFFPLDVYNNTTLDANKSQ--------------AVTPLNSTPKDSHAT-
          	    .*   .   *.*: : . .   :  .               ...  :**   ::. 

MG02569.1 	LYSYSNSLGSEPGVWLGDPG--SYSCGETPPATTVGIRAPRNHHHSAGEVDYTQSLSTSP
NCU02112.1	-AIYSESPGIEDVVALLEPTPNSFSAGETP---------PKMSRDHLREGDQTQSLSTSP
FG08557.1 	-VHYAGSQASDDGIWAGDAASNSYSCGETP---------PNRFKGYLGDVEAPPSVSTSP
AN8701.1  	--PGAQSSGRSSFELWRVGTTPPYSAGTTPP------LLSRTSGLGIERRAVTQHASLAA
          	    : * . .         . .:*.* **.        ..           .   * :.

MG02569.1 	NTRIAGFRRSNSAIAAAPGLAASLPRTLHNIV-SSSPPDGPTRKRVSPAEILG-NLAP-A
NCU02112.1	SNRL--FTRSNTAVSA--AFS--LPKTLAGMV-SGSPPDPPPRKRPSPAEAIFNNLAP-S
FG08557.1 	NTRF--LNRANSGLAS--AFAANLPRSFAGIG-SSSPPNHG-RKKPSPAETYLSSLAPGN
AN8701.1  	SPDQQAQPRSSSGFGS--ALASSLTRSFNTFAPSSASPPAGDLLRRKHAQVESPRTAVAS
          	.    .  *:.:...:  .:::.*.:::  : .*.:.*      : . *:      * . 

MG02569.1 	VTWGASTVFGAGAAARHADAAAGTAAVAAELRAAAAAASVAGTRTPSTDGIDAPYTHLES
NCU02112.1	VTWGTSTVFGGTAPE------------------------TPGTARTSLSDDEGRNHDMTV
FG08557.1 	VTWSASTTMDSNG------------------------------GRTSLDDDEFRRDDFLP
AN8701.1  	GTWTPSITTPRPASTNN----------------DHLTPAVISSLQTHSDTESERLTPKNA
          	 ** .* .    ..                      :.:  .:  .  .  .        

MG02569.1 	MVAVSVSVKLFNESSFDDDGWMTTPFLEPKLGALYASYRHAYAEMLQVWGQPLARLEIMK
NCU02112.1	LVPVAVSVVPENQGLFDDDGWLNTPLLDPSRNHVYSYYRYAYAEMLQMWGQPLSRLEIMK
FG08557.1 	LVPTKISVTMEDQTVFDDDGWLNIPLLESSQADAHARYRYAYAEMLQMWNQPLARLEIMK
AN8701.1  	KPTKKVRVTLKNKGAFDSDNATQALFLDPKKDSLYRSYRMAYAHLLSVWGLPIQRSEILK
          	  .  : *   ::  **.*.     :*:..    :  ** ***.:*.:*. *: * **:*

MG02569.1 	FNILKGGDRNPDSKSMFGQHNDDDEDGGHKDPQNSPHVRDTGATVKDEYGEEVVTGTPSP
NCU02112.1	FNVLKG--------------DKTSSSGCYSDVTHS----IDGCYDHETAENTAANSASQN
FG08557.1 	FNSLKE--------------DMT---GGHVDDNYS----ATG--HDGANGKIHGNHANSS
AN8701.1  	IGDLKQ------------PNHAPTREGGPSSPEDS-----------LSLPRPLRTTSPEI
          	:. **               .    .*   .   *               .   . : . 

MG02569.1 	TLTVSKRRHLKTLMGSGRGLDVTGICRIHETNLEPLQTSGRPLPSHYGSELVSIDGSTAE
NCU02112.1	PAFGGRKEQLQALIASGRGLDVTGICRIHEEQLDPVEYT-----------------RPTK
FG08557.1 	PIAMGKKDQLQALLASGRGLDVTGMCRIHEIQLEPLRYT-----------------SSDP
AN8701.1  	SADKDGKGLGIQRHCIGCGAPQLSIFAIQTTVDAALAAR------------------KSK
          	.   . :         * *    .:  *:     .:                        

MG02569.1 	MTIGGAVGTCERCQHS----LQSQLRCVYCAEPVAALYPACLGCGCPSHEKCLAEWHAAG
NCU02112.1	GKYGGAVGTCHRCVKAGNKKEQKQLRCVYCLEPIAALYPPCLNCGCAFHEACLAEWHEMG
FG08557.1 	-KVGGAVGTCDRCHQT-----QSQLTCVYCLEPVDALFPPCLACGCASHEACLAEWHAAG
AN8701.1  	TKGDATSITCLKCKPTR--RLPLKLPCAICHEVVEGMLTPCLGCGHVCCFSCYRDWLSVA
          	 . ..:  ** :*  :       :* *. * * : .: ..** **      *  :*   .

MG02569.1 	ERLCPAGDECDCGSEADNGKIESWAAIQAALLSARKAEKERQAAATAARGRKTVRVAGGP
NCU02112.1	EEECPAGDDCCCVEDASTAHVERWSALQAAVAAMNKMRSAASGSLHQQVDRDKEKSSGNQ
FG08557.1 	ETQCPAGDECNCAEEAADGQVESWAALQGAMLKGQRYLMMLPGPAMEDSDED--------
AN8701.1  	STDPTQQHSDDSDQEPNSQFCPSGCGCKCADHGMTDIVASSPLAWSRASSPVHEGDSSNP
          	.   .  ..  . .:.        .. : *             .     .      :.. 

MG02569.1 	PAMLNLPREAGIAGFITPPMASPNAARPRRKSMPGG------------------------
NCU02112.1	SSISRRSSIKTVLTGVASVATGGTGSRPRRRSVPTDLAKFMRGGRSDPNAYRPNDDNDDD
FG08557.1 	------------------------AKAPR-------------------------------
AN8701.1  	------------------------ASAYRSQN----------------------------
          	                        .   *  .                            

MG02569.1 	------------------------------------------------------------
NCU02112.1	YDEDDDDLPTIAITSSNGGSCDRSRSAHPTNRRAVSTASPSGIITNTIPHQFGRRRGGSS
FG08557.1 	------------------------------------------------------------
AN8701.1  	------------------------------------------------------------
          	                                                            

MG02569.1 	------------------------------------------------------------
NCU02112.1	ASHLAATGGYSSGYGHRSSNLSHFYIAADDNSGDSADSNNEGTATTQFTASSNSNRASQT
FG08557.1 	------------------------------------------------------------
AN8701.1  	------------------------------------------------------------
          	                                                            

MG02569.1 	------IGLHEQEQHTQK---------QHHAG----------------------------
NCU02112.1	LLSGNDIDREEWESVASSRGIPSLSNDPLHPSHHSRSGSRSGNSSRSASGGSDTGGGKDN
FG08557.1 	----------DWERVES-----------MVPSRMQG------------------------
AN8701.1  	--------RREASRSIDG------------------------------------------
          	          : .   .                                           

MG02569.1 	------------------------------------------------DPVRRLREEEGE
NCU02112.1	TTTSSSSMTTTTPRVGDSGSDNSKRPPHHERTPSGRGFTALKPPPYGDEPISAARLSLGN
FG08557.1 	--------------------------------------------------FSAASISLGN
AN8701.1  	------------------------------------------------LVNPDHGHGHAE
          	                                                          .:

MG02569.1 	DGEEGMDREGWESVTLPPGG------------SGSGSGGTTTPAGSRLPSAAAHLNRLRK
NCU02112.1	RLKKHIAGSSAASGSGSKGGESGSGGNGGNAGSDSGGGGDTT---GVAGGSGGSSSSLLL
FG08557.1 	RLRKSAGEWSRAS---------------------SLRRGDRR---NIM------------
AN8701.1  	DDLEQWGASPFASLARGLGG--------------GLSRGLRGKEDRRKPGKAPGSSTFVP
          	   :        * :   ..              .   *     .    . .   .    

MG02569.1 	QVGEMTGRPSILRQRSGGVLQKKMP--
NCU02112.1	GGGGGAQRPSLLRKKSTGVAVWRGGGN
FG08557.1 	---------------------------
AN8701.1  	KRSSMNQVLS-----------------
          	  .      :
```
